# Editorial: Prophylactic efficacy and safety of COVID-19 vaccines in preventing disease caused by various SARS CoV-2 variants

**DOI:** 10.3389/fcimb.2024.1384601

**Published:** 2024-03-13

**Authors:** Haidar Abdul-Lateef Mousa, Mairaj Ahmed Ansari

**Affiliations:** ^1^ College of Medicine, University of Basrah, Basrah, Iraq; ^2^ Department of Biotechnology, SCLS, Jamia Hamdard, New Delhi, India; ^3^ Centre for Virology, SIST, Jamia Hamdard, New Delhi, India

**Keywords:** COVID-19, SARS-CoV-2, vaccination, prophylaxis, prevention, complications

The emergence of COVID-19 pandemic put the world into a challenge to control the illness. In the relentless battle against the COVID-19 pandemic, numerous vaccines has been developed and distributed to mitigate the spread of the SARS-CoV-2 virus. The virus’s undergoing continuous evolution, resulting in the appearance of various variants, it is crucial to assess the efficacy and safety of these vaccines in preventing infections within the constantly changing viral landscape. Within a relatively short period, several vaccines have been developed. There is no world consensus about vaccines approval. Each country has approved certain vaccines which might differ from other countries. The most widely employed vaccines were not extensively studied. The vaccines were used under emergency use authorization to battle the COVID-19 pandemic. The protective effect of these vaccines is required a continuous evaluation because the virus has continuous changes in its genetic sequence. The early data about the efficacy of the vaccines were mostly adopted by manufacturers. Therefore, independent studies are required for approved vaccines to verify the protective effects of different vaccines especially in cases of new viral mutations such as Delta and Omicron variants. Delta variant may cause more severe illness than the other variants whereas Omicron variant spreads more easily than other variants and is less severe in general. The virus proved to have the ability to develop mutations continuously whereas the available vaccines were not modified as fast as the viral pace. It is most likely that new variants will emerge that their virulence cannot be predicted. The emergence of highly virulent virus might escape the protective effects of the available vaccines. Furthermore, the old and recently approved antiviral agents for COVID-19 treatment are not fully evaluated that a comprehensive data about their efficiency is required. The objectives of this Research Topic are to assess the safety, efficacy, and preventive ability of different COVID-19 vaccines.

The current Research Topic including original and review articles. Various studies has been conducted to check the efficacy and safety of COVID-19 vaccines in preventing disease caused by various SARS CoV-2 variants. Ying-hao et al. carried out a retrospective study including 25207 cases with SARS-CoV-2 Omicron variant infection of whom 81.1% were fully vaccinated. They found that the predictors for the illness deterioration were comorbidity, fever, age, cough, fatigue, and taste disorders. A meta- analysis study by Voleti et al.on myocarditis in SARS-CoV-2 infection vs. COVID-19 vaccination. They aimed to compare the incidence of myocarditis in COVID-19 vaccinated people and in severe acute respiratory syndrome-coronavirus 2 (SARS-CoV-2) infection groups. They made a conclusion that risk of myocarditis is more than seven fold higher in persons who were infected with SARS-CoV-2 as compare to the vaccinated individuals. There is debate on the safety of COVID -19 vaccine on adults, a study investigated the serious adverse events reported for COVID-19 vaccines in adolescents and young adults by Cappelletti-Montano et al. They identified a risk of serious adverse events following COVID-19 vaccination markedly higher compared to Influenza and HPV vaccination, both for teenagers and young adults. A review on Omicron related COVID-19 prevention and treatment measures for patients with hematological malignancy and strategies for modifying hematologic treatment regimes conducted by Guo et al. They explained that individuals with hematological malignancy and COVID-19 remain susceptible to severe infection and mortality, especially those with chronic lymphocytic leukemia and those undergoing chimeric antigen receptor T-cell treatment. They also recommended that patients with hematological malignancy should be assessed properly by hematologists before chemotherapy or immunosuppressive treatment, providing the COVID-19 vaccine with proper dosage was administered.

## Author contributions

HM: Writing – original draft, Writing – review & editing. MA: Writing – original draft, Writing – review & editing.

